# Utility of the 3Di Short Version for the Diagnostic Assessment of Autism Spectrum Disorder and Compatibility with DSM-5

**DOI:** 10.1007/s10803-016-2713-9

**Published:** 2016-01-29

**Authors:** Geerte Slappendel, William Mandy, Jan van der Ende, Frank C. Verhulst, Ad van der Sijde, Jorieke Duvekot, David Skuse, Kirstin Greaves-Lord

**Affiliations:** Department of Child and Adolescent Psychiatry/Psychology, Erasmus Medical Center-Sophia Children’s Hospital, Wytemaweg 8, 3015 CN Rotterdam, The Netherlands; Yulius Mental Health, P.O. Box 753, 3300 AT Dordrecht, The Netherlands; Division of Psychology and Language Sciences, Faculty of Brain Sciences, University College London, 1-19 Torrington Place, London, WC1E 7HB UK; School of Life and Medical Sciences, University College London, 30 Guilford St, London, WC1N 1EH UK

**Keywords:** 3Di, Autism spectrum disorder, DSM-5, Validity, Factor analysis, Assessment

## Abstract

The Developmental Diagnostic Dimensional Interview-short version (3Di-sv) provides a brief standardized parental interview for diagnosing autism spectrum disorder (ASD). This study explored its validity, and compatibility with DSM-5 ASD. 3Di-sv classifications showed good sensitivity but low specificity when compared to ADOS-2-confirmed clinical diagnosis. Confirmatory factor analyses found a better fit against a DSM-5 model than a DSM-IV-TR model of ASD. Exploration of the content validity of the 3Di-sv for the DSM-5 revealed some construct underrepresentation, therefore we obtained data from a panel of 3Di-trained clinicians from ASD-specialized centers to recommend items to fill these gaps. Taken together, the 3Di-sv provides a solid basis to create a similar instrument suitable for DSM-5. Concrete recommendations are provided to improve DSM-5 compatibility.

**Electronic supplementary material:**

The online version of this article (doi:10.1007/s10803-016-2713-9) contains supplementary material, which is available to authorized users.

## Introduction

Diagnosing autism spectrum disorder (ASD) is a complex process that requires standardized collection of information through both child observations and parental interviews, as well as other information concerning the functioning of the child (Falkmer et al. 2013; Ozonoff et al. 2005). Standardized and validated instruments are available to aid professionals in this process, but can be costly to administer, since they are often time consuming and require specific expertise to correctly administer and interpret. While the use of standardized parental interviews is becoming more and more common in specialized centers (Ashwood et al. 2014), only 50 % of UK child development teams make use of them (Palmer et al. 2010). One of the main reasons for this relates to feasibility, i.e. the required time investment (Matson et al. 2007). Standardized parental interviews, such as the ADI-R or DISCO (Rutter et al. 2003; Wing et al. 2002), commonly require up to 3 h to administer. This constitutes a significant time burden on both parents and clinicians.

To meet this need for a clinically feasible standardized and valid parental interview, the Developmental Diagnostic Dimensional Interview-short version (3Di-sv; Santosh et al. 2009) was developed. This is a 45-min version of the original 3Di (Skuse et al. 2004). Like its longer equivalent, the 3Di-sv is a computerized parental interview for ASD assessment. It offers dimensional scores on the three pervasive developmental disorders (PDD) domains of social reciprocity, communication and repetitive and stereotyped behavior (RSB), as defined in the DSM-IV-TR (American Psychiatric Association 2000), as well as PDD classifications based on validated cut-off scores on these domains. Items were selected based on existing 3Di research data, and scores and classifications from the shorter interview showed excellent agreement with those on the longer version as well as ADI-R classifications (Santosh et al. 2009). This original study found sensitivity and specificity values of over .85 when comparing 3Di classifications to clinical diagnoses.

While these results are promising, it should be noted that they were based on existing data obtained from the full interview, which were then rescored based on the new algorithm. Consequently, scores on the selected items might have been influenced by information obtained by asking additional questions as part of the longer interview that were subsequently removed in the shortening process. The validity of the 3Di-sv as a stand-alone instrument was supported by a study in Thailand (Chuthapisith et al. 2012), which resulted in fair to good areas under the ROC curves for all three scales compared to clinical DSM-IV-TR diagnoses (.79–.89). Lai et al. (2014) found excellent (.95) sensitivity for the Chinese translation of the 3Di-sv and fair specificity (.77). For the Dutch version, preliminary explorations pointed towards similar results (Slappendel et al. 2013), with moderate sensitivity (.60) and fair (.75) specificity compared to clinical DSM-IV-TR diagnosis, and moderate to strong correlations (.25–.65) between 3Di-sv domain scores and scores on the Social Responsiveness Scale (SRS; Constantino and Gruber 2005).

So far, research on the validity of the 3Di-sv as a standalone instrument has been performed using typically developing children (Chuthapisith et al. 2012), or children referred for symptoms unrelated to ASD (Lai et al. 2014) as controls. This is not representative of the reality of clinical work, where ASD specific instruments will not be used unless a child shows elevated levels of ASD symptoms according to parent and/or teacher report. Using controls who show no reasons for suspicion of ASD likely inflates sensitivity and specificity scores, by including children that would not normally be tested. Therefore, this study only included children with elevated levels of ASD symptoms as reported by parents on the SRS, indicating that parents feel their child shows significant ASD symptoms.

Beyond the regular validation concerns, the recent change from DSM-IV-TR to DSM-5 poses extra challenges. The DSM-5 draws on an increasing amount of research on the symptom domains of ASD (e.g. Boomsma et al. 2008; Frazier et al. 2008; Mandy et al. 2012; Snow et al. 2009; van Lang et al. 2006), which has led to a reformulation of the diagnostic requirements for ASD. The model of ASD has been changed from three dimensions to a two dimensional model that merges the reciprocity and communication domains into one social communication domain. Additionally, the RSB domain has been expanded to include both stereotypical communication and sensory hyper and/or hyporeactivity. These changes have prompted the developers of several ASD assessment instruments to revise both their item content and their scoring algorithms to better address these new criteria (e.g. Carrington et al. 2014; Kent et al. 2013; Lord et al. 2012). In doing so, the need to expand this research to the 3Di has already been expressed (Carrington et al. 2014). While studies have been done to relate the full 3Di ASD interview to the DSM-5 model, with positive results (Mandy et al. 2012, 2014), the 3Di-sv has not yet been studied with this aim. This means little is known about whether its factor structure conforms to the DSM-5 ASD model, and whether its items adequately cover the new criteria.

In order to fill the above-mentioned gaps in the literature on the 3Di-sv, the current study has three main aims. Firstly, to assess the validity of the 3Di-sv as a standalone instrument in a sample of children at high risk for ASD (aim 1). Secondly, to determine, through confirmatory factor analysis (CFA), whether the 3Di-sv fits the two factor (DSM-5) structure of ASD as well as the three factor (DSM-IV-TR) structure (aim 2). Finally, we explored content validity to clarify whether the items of the 3Di-sv represent all DSM-5 ASD criteria and exemplars (i.e. construct under or overrepresentation) (aim 3a), and subsequently explored possibilities to overcome construct underrepresentation by collecting perspectives of a panel of ASD expert clinicians frequently using the 3Di (aim 3b).

## Methods

### Participants

This project uses data collected as part of the Social Spectrum Study, a prospective multicenter study on autistic traits in clinically referred children and their families. The study was approved by the local medical ethics committee and the participating mental health care centers (MEC-2011-078).

The Social Spectrum Study focused on children aged 2.5–10 years with a clinical referral to mental health care. For this purpose, we selected consecutive referrals from six participating mental health care centers from both rural and urban areas in the south-west of the Netherlands during a 6 months interval at each site, across the period of April 2011–July 2012. While children were referred for a variety of mental health problems, we oversampled children with a high likelihood of having ASD. For this purpose, we used scores on the Social Responsiveness Scale (SRS; Constantino and Gruber 2005), which was completed by parents and teachers as part of routine clinical evaluation before intake. For the study, we then selected all children with a positive screen based on the parent report SRS (cut-off: total raw score ≥75; n = 428) and a random selection of children with a screen negative result (total raw score <75 on parent report SRS; n = 240) for further assessment, including the 3Di-sv and Autism Diagnostic Observation Schedule-2 (ADOS-2; Lord et al. 2012). Out of 668 invited families, 320 chose to participate in at least one part of the study. Written consent was obtained for all assessments.

3Di-sv data were available for 282 children. These cases did not differ significantly from non-participants regarding age (M = 6.7, SD = 2.3 vs. M = 6.9, SD = 2.3, *t*(666) = 1278, ns), gender (72 % male vs. 70 % female, χ^2^(1) = 453, ns) or total IQ (M = 95.8, SD = 17.4 vs. M = 93.0, SD = 16.9, *t*(493) = −1840, ns). SRS scores were higher for children for whom a 3Di-sv was available (M = 83.39 SD = 28.95 vs. M = 75.13 SD = 29.79, *t*(666) = −3.580, *p* < .001). For the current study, the sample was limited to only those children (n = 198) who scored above the raw SRS cut-off of 75. This ensured a sample that was comparable to the population in which the 3Di-sv might clinically be used, i.e. children with an indication of suspected ASD as indicated by parent-report, and excluded children without clinical levels of ASD symptoms, for whom the structure of ASD traits might be different (Mandy et al. 2014). The final sample had an average age of 7.55 years (SD = 2.56, range 2–12) when the 3Di-sv was performed, an average total IQ of 96.3 (SD = 17.47) and comprised of 73 % males. Of the participating children, 50 (25 %) had a clinical ASD diagnosis that could be confirmed with the ADOS-2. Out of the children who did not have an ADOS-2-confirmed ASD diagnosis, 30 % had an unconfirmed ASD diagnosis, 31 % had ADHD, 17 % had unspecified childhood disorders, 4 % had an anxiety disorder, 3 % had relational problems, and 9 % had another diagnosis. Finally, 4 % had no diagnosis on axis I, and 1 % had a deferred diagnosis and diagnosis was unknown for 2 %.

### Measures

#### Social Responsiveness Scale (SRS)

The parent-reported SRS (Constantino and Gruber 2005) was used to screen for ASD symptomatology. It contains 65 items that are scored on a 4-point scale from 0 (not true) to 3 (almost always true). The total score of the 65 items, which can range from 0 to 195, is used for screening purposes. A higher total score reflects more social impairment. The total score can be converted to a *T*-score, based on norms for gender and rater type, but to increase comparability between research studies, it is recommended to use the raw total score for research (Constantino and Gruber 2005). In the present study, the total raw cut-off score of 75 on the parent report SRS was chosen to indicate ‘high-risk’ for ASD, which was found to differentiate between children with ASD and children with other psychiatric disorders with a sensitivity of .85 and a specificity of .75 (Constantino and Gruber 2005). We used the Dutch translation of the SRS, which has been shown to have good psychometric properties (Roeyers et al. 2011).

#### Developmental Diagnostic Dimensional Interview-Short Version (3Di-sv)

The 3Di-sv is a 45-min standardized and computerized parental interview for ASD assessment (Santosh et al. 2009). The Dutch translation of the 3Di-sv was used. The Dutch 3Di is a direct translation of the English version. Items were translated by a Flemish psychiatrist (Wouter de la Marche, see De la Marche et al. 2015) and the first author of the present study, a Dutch psychologist, and cross-checked for appropriateness in both language areas as well as correctness of the translation, in regular consultation with the developers of the English 3Di and Dutch users. Items in the short version were independently back-translated and checked against the English 3Di-sv, after which adaptations were made where necessary. The Dutch translation was then programmed to enable computerized delivery and scoring.

The 3Di-sv scale scoring algorithm consists of 53 items, that constitute the domains of Reciprocal Social Interaction (24 items), Communication (21 items), and RSB (8 items). The algorithm items are averaged into subscales, which are then summed into scales that add up to create scores on the three DSM-IV-TR domains. An overview of the domains, scales and items is shown in Table 1. The 3Di-sv includes an additional 8 items on language development and age at first symptoms, which can be used to determine the classification of DSM-IV-TR PDD subtypes. However, in the current study, all PDD subtypes were combined into one category in line with the DSM-5 conceptualization of ASD, and thus items on language development and age at first symptoms were not used. Otherwise, cut-offs were unchanged from those defined by Skuse et al. (2004).

**Table 1:** Structure of 3Di-sv DSM-IV-TR algorithm

Domain	Scale	Items
Reciprocal Social Interaction	Use of non-verbal social cues (S1)	248: catching eye across room
		709: avoids eye contact
		710: looks away, seems inattentive
		249: smile in greeting when approaching
		251: smile in greeting when parents have been away
		252: smile in greeting when meeting
		someone outside home
		260: look surprised
		261: look guilty
		264: look embarrassed
	Peer relationships (S2)	369: play with children outside family
		331: joining group games
		655: rigidity when playing with peers
		347: invited to other children’s homes
		349: invites other children home
		237: regarded as rude
		717: perceived as odd and avoided
	Lack of shared enjoyment (S3)	304: joining in other people’s excitement
		303: spontaneous sharing of interests or activities
		299: sharing treats
	Lack of socio-emotional reciprocity (S4)	743: use other’s body as tool
		223: recognize emotion from tone of voice in the home
		224: recognize emotion from facial expression in the home
		624: recognize emotion from tone of voice outside the home
		309: recognizing subtle emotion
		706: doesn’t start conversations
		269: inappropriate smiling or laughing
Communication	Conventional gestures (C1)	279: clap for “well done”
		280: finger to lips
		282: beckon
		285: shake head for no
		737: joint attention
		742: nod to show is listening
	Conversational exchange (C2)	705: ignores conversational cues
		744: pre-verbal babbling conversations
		747: small talk
		379: talk about things no one’s interested in
	Stereotyped, repetitive or idiosyncratic speech (C3)	695: favorite phrases
		696: says things he doesn’t understand
		749: neologisms
		675: pronominal reversals
		702: understanding polite behavior
		703: embarrassing or tactless remarks
	Socio-emotional reciprocity (C4)	331: joining group games
		751: imitation
		338: fantasy play
		339: comments on play
Repetitive and stereotyped behavior	Restricted interests or preoccupations (R1)	723: one or more overriding interests
		754: odd or bizarre preoccupation
	Nonfunctional routines or rituals (R2)	750: endlessly exactly repeating a phrase
		756: precise odd rituals
	Stereotyped and repetitive motor mannerisms (R3)	766: hand or finger mannerisms
		767: whole body movements
	Preoccupations with part-objects or non-functional elements of materials (R4)	755: organizing toys
		757: unusual interest in taste, smell or feel

*3Di-sv* Developmental Diagnostic Dimensional Interview-short version, *DSM-IV-TR* Diagnostic Statistical Manual, Fourth Edition, Text Revision

Please note that whilst this version was developed in the original study by Santosh et al. (2009), there are minor differences between the version finally published in that paper, and the one implemented in the 3Di software program, and used in both this study and the study by Chuthapisith et al. (2012). However, differences between the two versions in terms of validation and reliability results and individual outcomes are negligible (W. Mandy, personal communication, June 28th, 2014).

All interviewers had at least a bachelor’s degree in a relevant field (such as medicine or psychology), were familiar with ASD, and received a day of formal training in the scoring and interpretation of the 3Di-sv by licensed trainers.

#### ADOS-Confirmed Clinical Diagnosis

For the clinical diagnosis of each child, diagnostic information was collected from the electronic patient files at all participating centers. These diagnoses were based on multidisciplinary diagnostic procedures at participating centers according to DSM-IV-TR criteria, and were independent of 3Di-sv classifications from our research. Diagnostic procedures at participating centers included a parental interview on the child’s early developmental history, medical history, and the child’s current functioning and an observation of the child during a semi-structured situation. A clinical diagnosis of ASD was coded as 1, a non-ASD diagnosis was coded as 0.

Subsequently, these clinical diagnoses were confirmed using the ADOS-2 classifications. The ADOS-2 (Lord et al. 2012) is a semi-structured child observation for the assessment of children’s social interaction, communication, play and imaginative use of objects, and is commonly used as part of the assessment of ASD. The ADOS-2 consists of four different modules based on age and expressive language level. The ADOS has shown good reliability, with interrater agreement on items averaging between 88.2 and 91.5 % depending on module, with interrater agreement on classifications ranging from 92 to 98 %. Predictive validity for all modules in our age groups ranges from adequate to good for non-autism ASD versus non-spectrum, and good to excellent for autism versus non-spectrum comparisons. The current study used modules 1 through 3. The ADOS-2 was performed and coded by trained and certified professionals. Classifications on the ADOS-2 were coded as 0 = non-ASD and 1 = ASD. Finally, scores on clinical diagnosis and ADOS-2 classifications were combined; thus cases who received a clinical diagnosis of ASD that was confirmed with an ADOS-2 ASD classification were coded as 1, all others as 0.

This ‘ADOS-2-confirmed clinical diagnosis’ was available for 146 children, of whom 50 (34.2 %) were considered to have ASD. These 146 participants did not differ from the larger set of participants (n = 198) in terms of age (M = 7.73, SD = 2.24 vs. M = 7.02, SD = 2.63, *t*(196) = −1.883, n.s.), gender (72 % male vs. 75 % male, χ^2^(1) = .184, ns), IQ(M = 96.99, SD = 16.89 vs. M = 94.14, SD = 19.27, *t*(176) = −.922, n.s.) or SRS total score (M = 98.77, SD = 16.93 vs. M = 98.79, SD = 19.24, *t*(196) = .005, n.s.).

### Statistical Analyses

#### Criterion Validity (Aim 1)

Criterion validity was assessed by comparing 3Di-sv classifications to clinical diagnoses confirmed with ADOS-2, and calculating sensitivity and specificity and their 95 % confidence intervals. STARD checklist for reporting of studies of diagnostic accuracy (Bossuyt et al. 2003) is available as a supplement.

#### Factor Structure of the 3Di-sv (Aim 2)

In order to test the factor structure of the 3Di-sv, this study used CFA to first test the two versus three factor ASD models against the data, using the 3Di-sv subscales as manifest variables. For an overview of the domains and scales as currently used in the scoring of the 3Di-sv, see Table 1. CFA was conducted using MPlus 7.2.

The two models were tested as follows, in line with the analyses performed in Mandy et al. (2014):

1. A three factor (DSM-IV-TR) model, illustrated in Table 1, based on a triad of social reciprocity (S1, S2, S3, S4), communication (C1, C2, C3, C4) and RSB (R1, R2, R3, R4);
2. A two factor (DSM-5) model, illustrated in Table 2, based on the two domains of social-communication impairment (S1, S2, S3, S4, C1, C2) and RSB (R1, R2, R3, R4, C3).

**Table 2:** Factor structure for DSM-5 confirmatory factor analysis

Domain	DSM-IV-TR algorithm scale
Reciprocal Social Communication	Use of non-verbal social cues (S1)
	Peer relationships (S2)
	Lack of shared enjoyment (S3)
	Lack of socio-emotional reciprocity (S4)
	Conventional gestures (C1)
	Conversational exchange (C2)
Repetitive and stereotyped behavior	Restricted interests or preoccupations (R1)
	Nonfunctional routines or rituals (R2)
	Stereotyped and repetitive motor mannerisms (R3)
	Preoccupations with part-objects or non-functional elements of materials (R4)
	Stereotyped, repetitive or idiosyncratic speech (C3)

*DSM-5* Diagnostic Statistical Manual, fifth edition, *DSM-IV-TR* Diagnostic Statistical Manual, Fourth Edition, Text Revision

For the DSM-5 model, compared to the DSM-IV model, the social reciprocity and communication domains were merged into one domain, stereotyped an repetitive language use (C3) were moved to the RSB domain, and the social reciprocity (C4) scale was removed in order to remove the items on imaginative solo play, which are no longer included in the ASD criteria under DSM-5.

Since there is no one standard index of fit for CFA, we followed Byrne (2012) in reporting χ^2^, Root Mean Square Error of Approximation (RMSEA), Comparative Fit Index (CFI), and Standardized Root Mean Residual (SRMR). The χ^2^ statistics for CFA is based on the null hypothesis that the tested model is a good fit for the data; thus, lower χ^2^ values indicate better fit for the model. RMSEA is a measure of the expected fit of the tested model to the population. Lower values indicate a better fit, with values over .10 indicating poor fit, values between .10 and .08 indicating mediocre fit, .08–.05 indicating reasonable fit and values under .05 indicating a good fit. RMSEA is sensitive to sample size, and can underestimate fit in samples smaller than 250 participants (Hu and Bentler 1998). CFI is an incremental measure of model fit, which compares the fit of the current model to an unspecified baseline model. CFI is standardized to run from 0 to 1, with values over .90 indicating adequate fit and values over .95 indicating good fit. The SRMR indicates the standardized average residual after fitting the model to the data, ranging from 0 in case of perfect fit to 1. An SRMR value below .05 is generally considered to indicate a good fit, while values between .08 and .05 indicate adequate fit.

In order to be able to directly compare model fit between the DSM-IV-TR and DSM-5 models, we then re-ran the DSM-IV-TR model excluding the C4 scale (i.e. social reciprocity) that was dropped in the DSM-5 model. Fit on the models was then compared using the BIC. The BIC value gives an estimate of how well a model is likely to perform on a new dataset, and values can be directly compared between non-nested models provided the same variables are entered. Lower BIC indicates better fit, with a difference of 0–2 considered to be weak evidence of better fit of the model with the lower value, 2–6 positive evidence, 6–10 strong evidence, and more than 10 very strong evidence (Raftery 1995).

In order to further determine the internal consistency of the scales, Cronbach’s alpha was calculated for each of the domains of both the three and two factor models based on the individual item scores. Cronbach’s alpha values over .7 were considered adequate and values over .8 were considered good. Changes in alpha values when items were deleted were inspected in order to determine if any items were misspecified.

#### Construct Representation (Aim 3)

While the model we used for the factor analysis gives an indication of the fit of the 3Di-sv scales to the DSM-5 model, it offers no information on individual items and how well the different criteria are covered by the current content of the 3Di-sv. Therefore, in order to determine how well the 3Di-sv covers all symptoms of ASD as defined in the DSM-5 at an item level, firstly individual items part of the 3Di-sv were matched to the criteria and exemplars of the DSM-5 (aim 3a). In order to create a good match of items to the criteria, a three step procedure was followed, similar to that used by Kent et al. (2013). First, two researchers (GS and KGL) matched the items to DSM-5 criterion exemplars. This matching was then shared with a researcher (JD) and a clinician who were both well acquainted with the 3Di-sv for feedback. The adjusted matching was subsequently shared with clinician/researchers familiar with the 3Di (WM, DS), who were not involved in the set-up of the current study. Items on which there was disagreement were individually discussed to reach consensus on best fit. The list of exemplars with their matched items was then shared with all other co-authors for feedback. The matches were agreed upon by all authors. Finally, trained 3Di users in the Netherlands were contacted for feedback. They were asked to comment on the matching, leading to one item moving to a different scale.

In order to be able to propose additional items that might address construct underrepresentation (aim 3b), five experienced, 3Di-trained clinicians from specialized ASD centers filled out a questionnaire. This questionnaire consisted of items that were included in the longer DSM-5 route of the 3Di (i.e. 196 items) as recently developed for a new English version of the program (D. Skuse, personal communication, February 25th, 2015). Suggested items for the ‘insistence on sameness’ and ‘inflexible adherence to routines’ exemplars were taken from the 3Di DSM-5 subscales ‘adherence to routines’ and ‘resistance to change’, and clinicians were asked which of these two DSM-5 exemplars they best matched. Suggested items for hypo and hyperreactivity to sensory input were taken from the DSM-5 scales ‘sensory interest’, ‘hyposensitivity to sensory input’ and ‘hypersensitivity to sensory input’, and clinicians were asked whether these best matched ‘hyper and hyporeactivity to sensory input’ or ‘sensory interests’. Subsequently, for each item, they were then asked to rate how important on a 5-point Likert scale they felt the item was as an index for the scale they had selected. Finally, the researchers selected the items based on a unanimous agreement on its belonging to the required scale, as well as an average score of at least 4 for its importance.

## Results

### Criterion Validity

Crosstabs for 3Di-sv classifications and ADOS-2-confirmed clinical diagnoses are shown in Table 3. These numbers result in a sensitivity of .84 (CI .70–.92) and specificity of .54 (CI .44–.63). Given the relatively low specificity as compared to the existing literature (Chuthapisith et al. 2012; Lai et al. 2014), we decided to perform 3 post hoc analyses: Firstly, we checked whether results differed if we stratified our sample based on age (i.e. 2.5–6 vs. 6–10). These post hoc analyses showed that sensitivity and specificity for these groups did not differ from each other (sensitivity .80 [CI .44–.96] and specificity .55 [.31–.78] for children under age 6 vs. sensitivity .85 [.69–.94] and specificity .53 [.31–.78] for children over age 6). Secondly, we checked if results differed if we stratified our sample based on clinical setting (i.e. secondary, general mental health care vs. tertiary, specialized mental health care). These post hoc analyses again showed no difference between the groups (sensitivity .86 [CI .70–.95] and specificity .56 [.44–.67] for secondary centers vs. sensitivity .77 [.46–.94] and specificity .44 [.22–.69] for tertiary centers). Finally, we checked sensitivity and specificity in our full clinically referred sample (n = 282) since this better reflects the sample selection used in previous studies cited above. These post hoc analyses resulted in a sensitivity of .77 (CI .64–.87) and specificity of .67 (CI .59–.74).

**Table 3:** Cross tables for comparison of 3Di-sv classification to ADOS-2-confirmed clinical diagnosis for SRS-positive (n = 146)

	3Di-sv classification non-ASD	3Di-sv classification ASD
Clinical diagnosis non-ASD	51	45
Clinical diagnosis ASD	8	42

*3Di-sv* Developmental Diagnostic Dimensional Interview-short version, *PDD* pervasive developmental disorder, *ADOS* Autism Diagnostic Observation Schedule, *ASD* autism spectrum disorder

### Factor Structure of the 3Di-sv

Table 4 shows indices of fit for the ASD three versus two factor models. All fit indices show adequate fit for both the DSM-IV-TR and the DSM-5 model. Rerunning the DSM-IV-TR model without the C4 scale resulted in a BIC value of 7634.281, compared to a BIC of 7623.601 for the DSM-5 model. The difference in BIC of 10.7 constitutes very strong evidence for a better fit of the DSM-5 model than the DSM-IV-TR model according the standards for interpretation of BIC scores as published by Raftery (1995).

**Table 4:** Model fit indices for confirmatory factor analysis tested against screen positives based on SRS > 75 (n = 198)

Model	χ^2^	DF	RMSEA	CFI	SRMR	BIC
DSM-IV-TR	95.543	51	.065 (.044–.085)*	.927*	.056*	8369.978
DSM-IV-TR without C4 scale	70.649	41	.060 (.035–.085)*	.941*	.054*	7634.281
DSM-5	70.545	43	.057 (.031–.080)*	.945*	.058*	7623.601

*SRS* Social Responsiveness Scale, *DF* degrees of freedom, *RMSEA* root mean square error of approximation (<.08 suggests adequate fit, <.05 suggests good fit), *CFI* Comparative Fit Index (>.90 suggests adequate fit, >.95 suggests good fit), *SRMR* standardized root mean residual (<.08 suggests adequate fit, <.05 suggests good fit), *BIC* Bayesian Information Criterion (lower values indicate better fit), *DSM-IV-TR* Diagnostic Statistical Manual, Fourth Edition, Text Revision, *DSM-5* Diagnostic Statistical Manual, fifth edition

* Adequate fit

In line with the adequate fit indices for the CFA, Cronbach’s alpha values generally pointed towards adequate internal consistency for the scales. For DSM-IV-TR, the values were .86 for social reciprocity, .76 for communication, and .64 for RSB. For the DSM-5 domains, Cronbach’s alpha for social communication reached .88, and .71 for RSB. The reader should note that since the subscales differ in the number of items, alpha values are not directly comparable between the scales. There were no misspecified items, as alpha values did not improve after removing any items.

### Construct Representation

Table 5 shows how 3Di-sv items were considered to match up to the new DSM-5 criteria and exemplars. Five items, addressing sharing of food, pronominal reversals, solo fantasy play and imitation, could not reliably be matched with any of the exemplars. Four exemplars of DSM-5 criteria were not represented by any of the 3Di-sv items: ‘insistence on sameness’, ‘inflexible adherence to routines’, and ‘hyperreactivity to sensory input’ and ‘hyporeactivity to sensory input’, and could be considered to be construct under representations.

**Table 5:** 3Di-sv items matched to DSM-5 ASD criteria and exemplars

DSM-5 criterion	Exemplar	Items in 3Di-sv
*A: Social communication*		
1: Social-emotional reciprocity	Abnormal social approach	237: regarded as rude
		717: perceived as odd and avoided
	Failure of normal back and forth conversation	744: pre-verbal babbling conversations
		747: small talk
		679: talk about things no one’s interested in
	Reduced sharing of interests	303: spontaneous sharing of interests or activities
	Reduced sharing of emotions or affect	223: recognize emotion from tone of voice in the home
		224: recognize emotion from facial expression in the home
		624: recognize emotion from tone of voice outside the home
		309: recognizing subtle emotion
		304: joining in other people’s excitement
	Failure to initiate or respond to social interactions	705: ignores conversational cues
		706: doesn’t start conversations
2: Nonverbal communicative behaviors used for social interaction	Poorly integrated verbal and nonverbal interaction	742: nod to show is listening
		737: joint attention
	Abnormalities in eye contact	248: catching eye across room
		709: avoids eye contact
		710: looks away, seems inattentive
	Abnormalities in body language	743: use other’s body as tool
	Deficits in understanding and use of gestures	279: clap for “well done”
		280: finger to lips
		282: beckon
		285: shake head for no
	Lack of facial expressions and nonverbal communication	260: look surprised
		261: look guilty
		264: look embarrassed
		249: smile in greeting when approaching
		251: smile in greeting when parents have been away
		252: smile in greeting when meeting someone outside home
3: Developing, maintaining and understanding relationships	Difficulties adjusting to social contexts	702: understanding polite behavior
		703: embarrassing or tactless remarks
		269: inappropriate smiling or laughing
	Difficulties sharing imaginative play	369: play with children outside family
		331: joining group games
		655: rigidity when playing with peers
	Difficulties making friends	347: invited to other children’s homes
	Absence of interest in peers	349: invites other children home
B: Restricted, repetitive patterns of behavior		
1: Stereotyped or repetitive behaviour	Motor movements	766: hand or finger mannerisms
		767: whole body movements
	Use of objects	755: organizing toys
	Speech	695: favorite phrases
		696: says things he doesn’t understand
		749: neologisms
2: Rigidity	Insistence on sameness	–
	Inflexible adherence to routines	–
	Ritualized verbal or nonverbal behavior	750: endlessly exactly repeating a phrase
		756: precise odd rituals
3: Restricted, fixated interests	–	723: one or more overriding interests
		754: odd or bizarre preoccupation
4: Sensory abnormalities	Hyperreactivity to sensory input	–
	Hyporeactivity to sensory input	–
	Sensory interests	757: unusual interest in taste, smell or feel
Unmatched items		338: solo play with miniatures
		339: comments on solo play
		675: pronominal reversals he/she
		299: spontaneous sharing of treats
		751: imitation of daily activities

*3Di-sv* Developmental Diagnostic Dimensional Interview-short version, *DSM-5* Diagnostic Statistical Manual, fifth edition, *ASD* autism spectrum disorder

Therefore, a panel of 5 experienced, 3Di-trained clinicians from ASD specialized centers completed a questionnaire on the items that could be added in order to improve the construct representation for these scales. Table 6 shows the items considered to be most representative of the scales based on the responses of these clinicians. All suggested additional items (n = 14) were unanimously considered to belong to these exemplars with an importance of at least 4 on a scale of 1–5.

**Table 6:** Items to be added to improve 3Di-sv construct representation based on clinician consensus

Exemplar	Item	Average rating (range)
Insistence on sameness	1212: insistence things remain in exact place	4.0 (3–5)
	1214: compulsive sorting	4.0 (3–5)
Adherence to routines	758: strict daily routine	4.4 (3–5)
Sensory hyporeactivity	1293: insensitive to discomfort or pain	4.2 (4–5)
	1295: does not notice outside temperature	4.4 (3–5)
Sensory hyperreactivity	99: current: hypersensitivity to everyday noise	4.2 (2–5)
	107: current: covers ears for normal noise	4.2 (2–5)
	111: adjust daily activities because of noise	4.2 (3–5)
	1290: hypersensitive to scent	4.2 (3–5)
	101: lifetime: hypersensitivity to everyday noise	4.6 (4–5)
	109: lifetime: covers ears for normal noise	4.6 (4–5)
	1230: avoids foods with different textures	4.6 (4–5)
	1277: picky about food	4.6 (4–5)
	1291: picky about fabric texture	4.6 (4–5)

## Discussion

In this study, we aimed to explore the utility of the 3Di-sv, by determining its validity, examining its DSM-5 factor structure, exploring its DSM-5 construct representation and examining ways to improve construct representation.

### Criterion Validity of the 3Di-sv

While the 3Di-sv showed good sensitivity compared to ADOS-2-confirmed clinical diagnoses, specificity was low. This low specificity for the 3Di-sv is atypical. Other studies (Chuthapisith et al. 2012; Lai et al. 2014) have found similarly high sensitivities, but found better specificity values. While we did a post hoc analysis to determine whether this might be explained by the selection of a sample of high ASD risk children, specificity in our full clinically referred sample was still low compared to other studies. The remaining difference may well be due to the stringency of our criterion. Lai et al. (2014) and Chuthapisith et al. (2012) both used clinical diagnoses as a criterion, not confirmed by any standardized assessment. Taking this approach would have increased the number of ASD-positive children in our sample, and thus would have increased specificity to be more in line with these other studies.

While results did not change when the sample was stratified by age or by type of center, the wide confidence intervals indicate that this may well be due to the remaining sample sizes after stratification; only 28 children under 6 were available for this analysis, and only 31 children from specialized ASD centers. Future studies might look more closely into these kinds of subgroups with larger samples, in order to learn more about the samples in which the 3Di-sv is most useful.

### Factor Structure of the 3Di-sv

Confirmatory factor analyses found a better fit against a DSM-5 model than a DSM-IV-TR model of ASD. The positive results for the DSM-5 model are in line with those from previous studies using the full version of the interview (Mandy et al. 2014, 2012), as well as studies using other measures to investigate the structure of the ASD phenotype (Boomsma et al. 2008; Frazier et al. 2008; Guthrie et al. 2013; Norris et al. 2012; Snow et al. 2009; van Lang et al. 2006). This suggests that the structure of the 3Di-sv is not changed by the removal of the extra items that make up the full interview, and supports the research from Santosh et al. (2009) suggesting that the 3Di-sv is a valid alternative for the full 3Di interview in situations where time restrictions are in play. Internal consistency of the preliminary DSM-5 domains was also confirmed by Cronbach’s alpha values.

### Construct Representation

Finally, while the underlying structure of the instrument may align well with the DSM-5, the 3Di-sv does not fully cover all symptom groups described in the new ASD criteria. Sensory hyper and hyporeactivity, inflexible adherence to routines and insistence on sameness are not adequately covered by the current question set of the 3Di-sv, and repetitive language use is scored under communication rather than RSB. This is likely to lead to under inclusion because some relevant symptoms are not recognized. In fact, sensory behaviors have previously been found to be particularly discriminative for ASD caseness (Carrington et al. 2014), making this an important gap to fill to create an optimally functional instrument.

In order to adapt the 3Di-sv to the DSM-5, we explored items that may be added to fill these gaps. Results from our questionnaire provide a good starting point for which particular items might be added, based on the perspectives of experienced, 3Di-trained clinicians specialized in ASD. However, in the longer run, we argue for an additional in-depth, data driven approach, based on data collected with the recently developed new DSM-5 3Di full version, using analytical techniques such as those used by the developers of the DISCO DSM-5 algorithm (Carrington et al. 2014) or the AQ-10 (Allison et al. 2012).

Another concern for the concept representation of the 3Di-sv are the items in the current version that are not linked to the DSM-5 exemplars. While it may seem that these items can simply be removed, more consideration should go into this decision. The DSM-5 criteria explicitly state that the exemplars listed are not to be considered exhaustive. This means that items that cannot be matched to a specific exemplar in the DSM-5 might still be considered good examples of behavior indicative of a limitation as intended by the DSM-5 criteria. This particularly seems to be the case for the items concerning the sharing of food treats, and imitative play. While neither fits closely to any of the defined exemplars, both do seem indicative of an understanding of social relationships and social reciprocity that could clinically be considered part of the criteria for the reciprocal communication domain as the DSM-5 defines it. This is less obviously the case for the items concerning solo fantasy play, which do not have a direct relation to social behavior. However, solitary fantasy play is one of many ways in which children practice social scenarios and how to behave in social situations, and thus could still be considered an important part of the process through which children develop adequate social skills (Hobson et al. 2013). Item selection approaches such as those mentioned above may well offer further guidance in deciding whether and, if so, where, these items should be retained.

### Methodological Considerations

Some methodological notes should be considered when interpreting the results of this study. For the validation of the 3Di-sv, we used ADOS-2-confirmed clinical diagnosis as a criterion. This standard combines clinical judgment, which is the gold standard for ASD diagnosis, with the clarity and replicability of standardized assessment. However, it does exclude children who were diagnosed with ASD but did not obtain an ASD classification on the ADOS-2 on the assumption that these are not true cases. The recommendation to combine child observation and parental interview for ASD diagnosis is made particularly because the results from the two sources complement each other, rather than overlapping perfectly. Thus, some of the false positives in our study may actually have been children with ASD who were missed by the ADOS-2.

For the CFA, we followed Mandy et al. (2014) in rearranging 3Di scales, rather than items. While this mostly works out well, it does lead to some concerns about specific items in the subscales. In particular, while the movement of the repetitive and stereotyped language scale to the RSB domain is in line with the changes in the DSM-5, this scale also includes items about issues such as asking inappropriate questions and mixing up pronouns, which may better fit the social communication domain, and thus may be misspecified on an item level. However, since the results of the Cronbach’s alpha calculations, which were done at an item level, support the idea that the factor analysis yielded two coherent domains, this does not seem to have unduly influenced the results. Conversely, even without changing the content of the scales, analyzing the same data on a different level—that is, using items or subscales rather than the scales used in our study—may change the results (e.g. De la Marche et al. 2015), so further analysis is needed to determine the best factor structure for the data on an item level. The current dataset lacks the power to perform the CFA with the larger number of variables this would involve. CFA should therefore be repeated within a larger dataset, so individual items can be entered into the analysis.

Finally, construct representation was based purely on a theory-driven, face validity perspective. In the future, it would be a valuable addition to determine which items would best add to the 3Di-sv in order to construct a route that fully represents the DSM-5 based on a data-driven procedure.

### Conclusion and Clinical Implications

The 3Di-sv, in this study, appears to be somewhat over inclusive in its classifications. While the confidence intervals for the sensitivity and specificity of the 3Di-sv in specialized ASD centers were wide, resulting in the differences with general centers being non-significant, the values do seem to indicate that the 3Di-sv may perform better in general centers with a less complex or severe population. However, future research with larger samples will have to confirm whether or not this is indeed the case.

While the current results indicate that the 3Di-sv may be a solid basis upon which to build to create a similar route that is compatible with the DSM-5, creating a new DSM-5 version of the 3Di-sv will require some adjustments. First, items will need to be added to better cover newly introduced criteria, such as insistence on sameness and sensory abnormalities. The current full interview already contains some of the items needed to make this adjustment, but a data-driven approach to determine which items best represent the relevant criteria would be a valuable addition to the research on the 3Di. Secondly, decisions will need to be made on how to deal with those items that over represent the ASD construct under the DSM-5. Items concerning related symptoms that do not quite fit the exemplars may still add diagnostically important information. More research is needed to determine their usefulness in order to decide how best to deal with these items. Finally, the current scoring algorithm still leads to scores on DSM-IV-TR domains, with stereotypical language symptoms scored under communication rather than RSB. Thus, the scoring algorithm and format for final scores will need to be adjusted in order to represent the new ASD conceptualization.

Finally, while changes to the 3Di-sv may improve the specificity, any single instrument is likely to over or under identify ASD cases in some samples. Therefore, standardized parental interview information should always be complemented by alternative sources of information, such as direct observation and/or school report.

## Electronic supplementary material

Below is the link to the electronic supplementary material.

Supplementary material 1 (DOCX 25 kb)
